# Sinonasal cancers treatments: state of the art

**DOI:** 10.1097/CCO.0000000000000726

**Published:** 2021-03-23

**Authors:** Marco Ferrari, Ester Orlandi, Paolo Bossi

**Affiliations:** a Section of Otorhinolaryngology-Head and Neck Surgery, Azienda Ospedaliera di Padova, University of Padua, Padua; b Radiation Oncology Clinical Department, National Center for Oncological Hadrontherapy (Fondazione CNAO), Pavia; c Department of Medical and Surgical Specialties, Radiological Sciences and Public Health – Medical Oncology, ASST-Spedali Civili, University of Brescia, Brescia, Italy

**Keywords:** chemotherapy, radiation therapy, sinonasal cancer, surgery

## Abstract

The present review provides the reader with the state-of-the-art concepts of sinonasal oncology in view of the latest literature data.

**Recent findings:**

Most recent publications in sinonasal oncology assessed treatment timing, centralization, surgical approach, margin status, orbit/neck management, salvage strategies, emerging surgical technologies, intensity-modulated radiation therapy (IMRT), volumetric modulated arc therapy (VMAT), particle radiotherapy, and neoadjuvant chemotherapy.

**Summary:**

Indications to endoscopic surgery for sinonasal cancer have plateaued and are unlikely to further expand. Endoscopic surgery provides noninferior results compared to open surgery and best suits timing constraints imposed by multimodal treatment. Management of orbit-encroaching sinonasal cancer is remarkably improving mostly owing to optimal use of nonsurgical strategies. Prognostic value of the margin status and management of the nodal basin and recurrent sinonasal tumors are far from being fully elucidated. Most promising surgical technologies are surgical navigation, optical imaging, and radiofrequency-aided ablation. IMRT and VMAT have theoretical technical advantages that are in the process of being clinically demonstrated. Pieces of evidence are progressively confirming the physical and radiobiological advantages offered by particle radiotherapy. Systemic therapy is being tested mostly in the neoadjuvant setting with the aim of improving outcomes in locally advanced sinonasal cancers; response to induction chemotherapy could better select a further locoregional approach.

## INTRODUCTION

Sinonasal cancers (SNCs) are very rare and heterogeneous diseases, whose histology may be represented by epithelial, soft tissue, bone and cartilage, hematolymphoid, neuroectodermal, germ cells, and secondary tumors. Even within the same histologic group, the rarity of the disease does not allow to reach a high consensus concerning the proper therapeutic approach. Current strategies come from retrospective studies and population-based registry analyses. Overall, the primary management for patients with locally advanced SNCs depends on the histological type, the pathway of local and regional diffusion, the performance status of the patient, the anticipated toxicities of the treatment, and the availability of a multidisciplinary and experienced team. In the majority of cases, the therapeutic strategy relies on the combination of surgery, radiotherapy (RT), and chemotherapy [1,2]. For unresectable disease or inoperable patients, definitive RT is proposed, often with concurrent chemotherapy to improve outcome and preserve organ function [1–3].

Diagnostic procedures, in terms of morphological and functional imaging, have increased the possibility to better delineate the primary tumor extension, as well as the pattern of nodal and distant metastatic status. Similarly, the integration of morphological, immunohistochemical and molecular evaluations allow the pathologist to increase the quality of histological report and the differentiation among similar histotypes.

In the past year, we have witnessed several publications on improvements in the surgical approach, RT techniques and systemic treatment integration for locally advanced SNCs, which will be summarized in the present paper. The main objective of clinical research in this disease aims to find therapeutic strategies able to improve outcome without jeopardizing the toxicity profile of treatments. We will discuss about therapeutic approaches mainly in epithelial SNCs. 

**Box 1:**
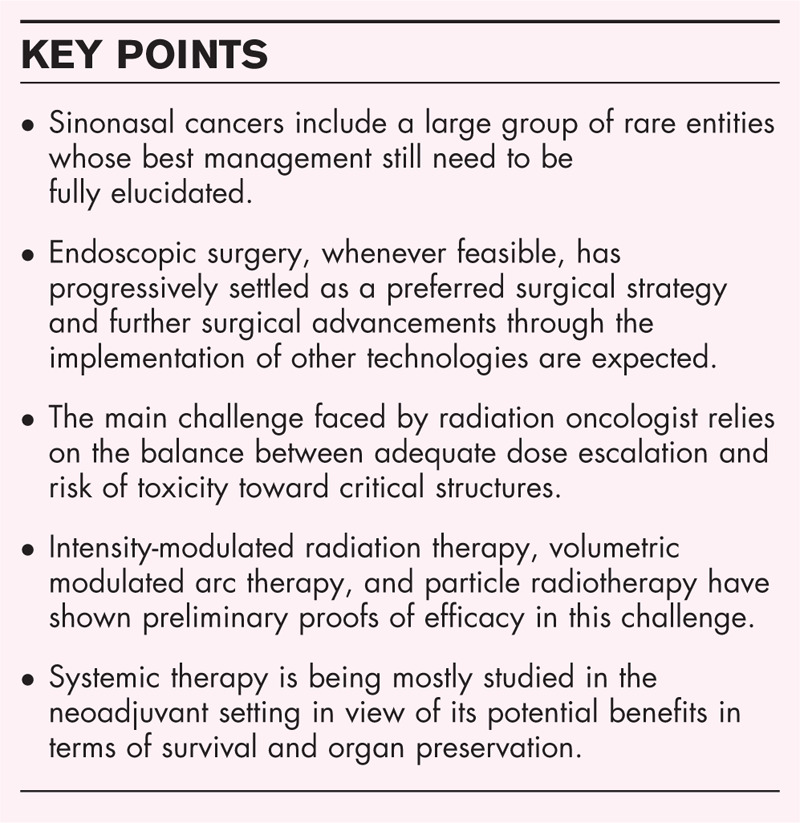
no caption available

## SURGICAL TREATMENT

Current surgical treatment of SNCs follows a relatively simple principle: endoscopic resection is indicated whenever all tumor boundaries can be adequately and safely controlled through a transnasal perspective; otherwise, maxillectomy, rhinectomy, cranioendoscopic resection, Riedel's operation, craniofacial resection or their combination are indicated. Limits of endoscopic surgery for SNCs have been plateauing over the last decades, and, to date, relative and absolute contraindications have considerably narrowed, especially for nasoethmoidal tumors. Consequently, advancements and refinements in the surgery of SNCs are accordingly shifting toward other objectives. Most of the recent publications on surgery for SNC are based on the National Cancer Database (NCD). This research strategy was rewarded with otherwise unreachable numbers of evaluable patients. However, rarity, biological heterogeneity, and relentless renewal of classification of SNCs make NCD data potentially fragile and mandates cautious interpretation.

### Timing and centralization of treatment

Two recent NCD-based publications highlighted the essentiality of adequate treatment timing in patients affected by SNCs [4^▪^,^5^]. Particularly, Goel *et al.*[4^▪^] analyzed 2267 patients and demonstrated that surgery-to-radiation interval alongside the duration of RT should be respectively maintained within 64 and 51 days to avoid survival worsening. Interestingly, the diagnosis-to-surgery interval did not independently affect survival, owing to the fact that patients sent to referral centers had the longest delay between diagnosis and surgery. Xiao *et al.*[5] presented a propensity score-matched (PSM) analysis demonstrating that endoscopic surgery is associated with a significantly shorter surgery-to-radiation interval irrespective of age, patient's income, comorbidity, facility type, and nodal status.

Teitelbaum *et al.*[6^▪^] (3835 patients) showed that patients affected by sinonasal squamous cell carcinoma (SCC) treated in high-volume centers (>16 patients/10 years) had a significantly better prognosis than those treated in low-volume centers (<12 patients/10 years) regardless of age, sex, T category, and treatment schedule. Of note, the majority (55.5%) of patients included in the study received a surgery-including treatment.

Overall, these data suggest that referring patients towards centers with adequate experience should prevail over rushing into therapy, whereas specific time constraints should be respected once treatment has begun. Endoscopic surgery, whenever feasible, could facilitate adhering to the optimal timing.

### Endoscopic versus open surgery

Recently, four papers assessing the different outcomes provided by open versus endoscopic surgery have been published. Among them, one was a single-institution study, which focused on sinonasal adenoid cystic carcinoma (30 patients) [7], and three were NCD studies, which focused on mucosal melanoma (686 patients) [8], olfactory neuroblastoma (ONB) (533 patients) [9], and non-SCC malignancies (1595 patients) [10], respectively. All these studies showed comparable survival outcomes when comparing the two techniques, with endoscopically treated patients benefitting from organ preservation [7] and shorter hospitalization [8,10] than those who underwent open surgery. However, the unplanned readmission rate was higher in patients treated through endoscopic resection for sinonasal mucosal melanoma, leading Farber *et al.*[8] to hypothesize a ‘potential tradeoff between the length of hospitalization and readmission rates’.

Overall, these recent publications reinforce the settled belief that well-performed endoscopic surgery ensures adequate oncologic outcomes, provided that it is accompanied with (neo)adjuvant treatments when indicated.

### Margin status

Three NCD studies assessed the role of margins in SNC over the last year. Torabi *et al.*[11^▪^] (2968 patients) and Jafari *et al.*[12] (7808 patients) focused their attention on sinonasal SCC. In the first study, treatment at a high-volume center was found to reduce the rate of positive margins, which were equally frequent in endoscopic versus open procedures. In the second study, the authors showed how tumors resected with macroscopically involved margins were associated with a similar prognosis compared to those treated with upfront nonsurgical strategies. PSM analysis showed that surgery yielding negative or microscopically involved margins was instead associated with proportionally higher overall survival (OS). Auger *et al.*[13] analyzed a series of 239 patients treated for sinonasal mucoepidermoid carcinoma, demonstrating that surgery with clear margins implies better outcomes as opposed to positive margins. Interestingly, they showed that adjuvant radiotherapy created a survival benefit only in patients receiving a complete resection.

These recent studies contributed to the awareness that achieving negative margins should be the goal of any surgery indicated for SNCs. The negative prognostic effect of margin involvement seems to depend on histology and the extent of residual disease.

### Orbit management

Management of SNCs encroaching the orbit is one of the most relevant challenge in organ-preservation treatments. Most relevant and recent literature on this topic includes a bi-institutional series of 163 SNCs and a single-institution series of 93 sinonasal SCC with orbit encroachment [14,15]. These studies demonstrated that deep orbital invasion, with special reference to the involvement of the orbital apex, is associated with poor outcome despite the aggressiveness of surgery. Promisingly, both studies concluded that neoadjuvant treatment offers a chance to spare the orbit even in patients with advanced orbital involvement. Patients who would have required an orbital ablation treated with neoadjuvant (radio)chemotherapy eventually received an orbit-sparing treatment in 62–75% of cases.

### Elective neck dissection

Two publications recently addressed the role of elective neck dissection (END) in patients affected by SNCs. Faisal *et al.*[16] published a systematic review with a meta-analysis of 255 patients treated for sinonasal undifferentiated carcinoma. They demonstrated that elective neck treatment decreases the risk of regional failure from 26.4 to 3.7% and advocated for END being the preferable strategy in view of the possibility to pathologically stage the neck, avoid irradiation-related morbidity, and reserve RT for a possible recurrence. Crawford *et al.*[17] performed an NCD-based study on 1120 patients treated for sinonasal SCC, out of which 220 received an END. They found that END was most frequently performed for maxillary sinus SCC, yet not producing any benefit in OS at multivariable PSM analysis, which included adjuvant RT as a covariate.

Indications alongside the best way to electively treat the neck in patients affected by SNCs remain debated. Laterality of treatment, addressability of retro- and para-pharyngeal nodes, frequent need for adjuvant treatment, and treatment-related morbidity are the main factors preventing firm conclusions. The histology-specific approach used in the recent literature might help getting closer to a consensus on how to electively treat the nodal basin.

### Recurrence and salvage surgery

Recurrence after surgery-including treatment and salvage therapy are among the most insufficiently investigated aspects of sinonasal oncology. Lehrich *et al.*[18] published an NCD-based analysis comparing 2804 patients treated with primary surgery versus 207 who underwent salvage surgery. They found that salvaged patients had a significantly lower survival probability than those primarily operated. Moreover, they found that stage and margin status were the only independent prognostic factors in patients treated through salvage surgery.

Knowledge of kinetics of relapse and biological behavior of recurrent SNC remains vague. Salvage surgery frequently implies a considerable risk of operation-related morbidity with a doubt benefit especially when multimodality treatment is hindered. Future research on sinonasal oncology will have the goal to identify the patients who might benefit from salvage surgery.

### Emerging technology in sinonasal oncologic surgery

The most relevant advancements in the field of sinonasal oncologic surgery can be clustered in technologies increasing the visualization of the tumor and ablative tools improving the ability to selectively hit the tumor while sparing nontumoral tissues.

Regarding the first group, surgical navigation and optical imaging are the most promising technologies. It has been recently demonstrated in a preclinical setting that facilitating the open ablation of tumors involving the maxillofacial skeleton through 3D rendering-based, real-time navigation provides a 20% gain in terms of adequate margin delineation [19]. Furthermore, Hart *et al.*[20] demonstrated in an animal model that a fluorescence endoscope optimized for near-infrared fluorescence detection is able of detecting tumors marked through intravenous injection of an anti-EGFR monoclonal antibody (panitumumab) conjugated to a fluorophore (IRDye800CW) with an adequate tumor-to-background ratio. These studies suggest that the implementation of technologies increasing the visualization of the tumor into surgical procedures for SNCs might be beneficial and will probably take place in the near future.

Endoscopic transnasal radiofrequency-ablation has been recently tested in a series of 97 patients, out of which 32 affected by a sinonasal or skull base malignancy [21]. The authors of this publication reported several advantages of radiofrequency ablation, the most relevant being optimal bleeding control, minimal thermal damage to adjacent neural structures (i.e. nerves and dura mater), and the ability to modulate the ablative effect of the instrument. Another ablative technology of potential interest is the intracavity photodynamic therapy through light emission into the sinonasal tract following systemic administration of a photosensitizer [22]. Despite the potential efficacy of this technology, a recent study demonstrated that further optimization is required prior to apply photodynamic therapy into the sinonasal cavity [23].

## RADIOTHERAPY

In advanced resectable tumors, surgery plus radiotherapy, with or without chemotherapy, is the most employed approach. In general, this applies to epithelial nonglandular and glandular cancers as well as mucosal melanoma, high-grade soft tissue sarcomas, chordoma, and high-grade craniofacial osteosarcoma. In all these clinical scenarios, adjuvant radiotherapy has been found to be effective in decreasing the incidence of local recurrence [24,25]. Unresectable cases are generally treated with definitive RT or radiochemotherapy [24].

Intensity-modulated radiation therapy (IMRT) allows improved dose distributions with lower doses to radiosensitive structures, thus reducing grade ≥3 toxicity rates by 5–35% compared to previous RT techniques [26] – in particular, blindness and optic neuropathy. However, no significant improvement in outcome has been reported so far, except for the recent paper by Moreau *et al.*[27] on 58 consecutive SNCs patients treated with postoperative volumetric modulated arc therapy (VMAT) to a total median dose of 63.7 Gy. Comparison with a retrospective historical cohort treated with 3D RT showed significantly improved local control (LC) and OS with VMAT. Another clinical strength of IMRT associated with systemic treatment is the management of a subset of T4 paranasal sinus and skull base cancers in order to preserve the organ function and avoid unacceptable sequelae [26].

In the field of high-precision therapy for SNC, particle (or charged particle) therapy, including protons and carbon ions, marks a new era. Compared to photon-based IMRT, particles have physical advantages with a sharp increase of the dose in a well-defined depth (the so-called Bragg peak) and a rapid dose fall-off that spares the healthy tissue distal to the tumor, leading to superior dose distributions. In addition, carbon ions, in light of their much higher radiobiological effectiveness, reduced oxygen enhancement ratio, and induction of clustered DNA damages, are potentially capable to effectively kill radio- and chemo-resistant tumors, such as adenoid cystic carcinoma, melanoma, adenocarcinoma, both in primary and recurrent presentation [28].

No prospective randomized trials have been published so far, because of the rarity of SNCs and the scarce availability of particle beam RT facilities. Recently, Zhang *et al.*[29^▪▪^] conducted a meta-analysis to compare the effectiveness of carbon ion RT (CIRT), proton RT (PT), and IMRT and estimate OS and LC in a real-world setting. The Authors analyzed a representative sample of 2282 patients from 49 cohorts (8 CIRT cohorts, 20 PT cohorts, 21 IMRT cohorts). The distribution of histological type was different according to the RT technique group: in the CIRT group, mucosal melanoma and adenoid cystic carcinoma were the most frequent histologies, whereas in the PT and IMRT groups, SCC was the main cancer. Most of the patients were treatment-naive, but there were 11.4%, 7.7%, and 10.9% of recurrent cases in CIRT, PT, and IMRT group, respectively. The 3-year OS and LC were significantly higher after CIRT than PT or IMRT. However, this meta-analysis suffers from major pitfalls, as the number of patients, the tumor sites, and the RT settings were quite different among groups. Moreover, the lack of toxicity data prevents clear conclusions on the advantage provided by CIRT.

After this meta-analysis, a handful of papers have been published. Yu *et al.*[30] reported the outcomes of 69 patients treated with PT between 2010 and 2016 by the multi-institutional Proton Collaborative Group. The most common histology was SCC, followed by adenoid cystic carcinoma. In total, 42 patients received *de novo* adjuvant irradiation (median total dose 58.5 Gy), whereas 27 received postoperative re-irradiation (median total dose 60 Gy). Overall, 30% of patients received chemotherapy. With a median follow-up of 26.4 months, the 3-year OS, disease-free survival (DFS) and locoregional control for *de novo* irradiation were 100%, 77.3%, and 92.9%, respectively. In the re-RT setting, the 3-year OS, DFS, and locoregional control were 76.2%, 32.1%, and 33.8%, respectively. No grade ≥3 late toxicities were reported.

Fan *et al.*[31] reported the outcome of 86 consecutive upfront treatment-naive and recurrent patients treated with PT between 2013 and 2018. The most frequent histology was SCC followed by adenoid cystic carcinoma. Total median dose was 70 Gy in the RT-naive cohort and 67 Gy for re-irradiated patients. Approximately 50% of patients received upfront surgery, more than 50% received chemotherapy. For naive patients, the 2-year LC, DFS, and OS were 83%, 74%, and 81%, respectively, whereas the corresponding percentages for re-irradiated patients were 77%, 54%, and 66% [29^▪▪^]. Overall, 6% of naive and 11% of re-irradiated patients had late grade 3 toxicities, including osteoradionecrosis, vision loss, and brain necrosis.

Pasalic *et al.*[32] reported physician-assessed toxicities and patient-reported outcomes in a prospective cohort of 64 patients treated with PT between 2011 and 2019. Most frequent histologies were ONB, sinonasal undifferentiated carcinoma (SNUC), and SCC. Approximately, 30% and 70% of patients received definitive RT (median total dose 66 Gy) and postoperative RT (median total dose 60 Gy), respectively. Overall, 70% of patients received chemotherapy (concurrent, neoadjuvant, or both). With a median follow-up time of 33 months, no late grade 3–4 neurologic physician-assessed toxicities were observed. Patient-reported outcomes suggested significant changes in the acute–subacute period but no long-term side-effects. The 3-year LC, DFS, and OS were 88%, 76%, and 82%, respectively [32].

A 10-year (2009–2019) experience from the University Hospital of Heidelberg showed the efficacy and safety of the primary and postoperative bimodal approach, including boost with CIRT and IMRT in 227 adenoid cystic carcinomas, to a median total dose of 80 Gy. With a median follow-up of 50 months, the 3-year LC rates were 79% for primary RT and 82% for postoperative RT, without significant difference, potentially due to the high number of macroscopic residue after surgery (R2). Significant worse adverse effects were observed in the postoperative setting, with 17% of late grade 3 toxicity [33].

Recently, a Japanese prospective study was conducted at Gunma University Heavy Ion Medical on locally advanced naive nasal cavity and maxillary sinus mucosa melanoma patients. Between July 2012 and January 2019, 21 patients underwent radical CIRT with a dose of 57.6–64.0 GyE in 16 fractions with concomitant and adjuvant chemotherapy with dacarbazine, nimustine, and vincristine. The 3-year local control rate was 92.3%. Eleven patients (52.4%) developed distant metastasis, which was the most frequent pattern of failure. No grade 3 or greater late toxicities were reported [34].

Finally, with regard to other histologies such as chordomas, chondrosarcomas, and other sarcomas, which are inherently radioresistant and frequently close to organs at risk of radiotoxicity, postoperative PT and CIRT may be particularly useful. Indeed, conventional photon RT is associated with a high rate of local failure and carries a significant risk of brainstem and cranial nerve damage. Recently, Iannalfi *et al.* reported on the prospective experience of the National Center for Oncological Hadrontherapy (CNAO) in Pavia, Italy, in treating through either PT or CIRT 135 patients with skull base chordomas who had received biopsy, incomplete, or complete gross resection [35]. With a median follow-up duration of 44 months, 5-year LC rates were 84% and 71% with PT and CIRT, respectively, without significant difference. The overall rate of severe (grades 3–4) toxicity was 12% and did not differ based on the type of particle used. Moreover, a recent NCD-based study on 863 patients with chondrosarcoma and 715 patients with chordoma treated with curative-intended conventional RT and PT from 2003 to 2014 reported that PT was associated with an improved OS at 5 years in a multivariate analysis [36].

## CURRENT TREATMENT PROTOCOLS AND INDICATIONS TO DIFFERENT TYPES OF SURGERY AND RT

Table 1 summarizes the most credited treatment schedules with curative intent a patient can be offered to date, based on literature analysis and authors’ personal experience.

**Table 1:** Most relevant sinonasal cancers

Histology	Highlights	5-year OS^b^	Treatment
Squamous cell carcinoma	Frequently located in the maxillary sinus Can arise de novo or ex inverted papilloma HPV-related multiphenotypic and nonkeratinizing variant might have pronounced radiosensitivity Low-to-moderate risk of nodal metastasis^a^	Intermediate	S + (C)RT nCT + CRT nCT + S + (C)RT
Intestinal-type adenocarcinoma	Frequently located in the nasoethmoidal complex Associated with wood-, leather-, or cork-working Heterogeneous geographical distribution Grade-dependent behavior Low risk of nodal metastasis^a^	Intermediate-to-high	S S + RT nCT + S + RT
Nonintestinal-type adenocarcinoma	Very rare ‘Wastebasket’ entity grouping diverse tumors Grade-dependent behavior	Intermediate	S S + RT
Olfactory neuroblastoma	Most often centered in the olfactory cleft Grade-dependent behavior Moderate-to-high risk of nodal metastasis^a^ Slow kinetic, nonnegligible rate of late relapse	High-to-very high	S S + (C)RT nCT + (C)RT nCT + S + (C)RT
Mucosal melanoma	Very aggressive tumor Noncontiguous mucosal extension (i.e. melanosis, satellites) Low-to-moderate risk of nodal metastasis^a^ High risk of distant metastasis^a^	Low-to-very-low	S + RT^c^ RT^c^
Adenoid cystic carcinoma	Tendency to submucosal, perineural, and lymphovascular spread Grade-dependent behavior High-grade transformation variant Slow kinetic, remarkable rate of late relapse Low-to-moderate risk of nodal metastasis^a^ High risk of distant metastasis^a^ High long-term disease-specific mortality	Intermediate-to-high	S + RT^c^ RT^c^
Sinonasal undifferentiated carcinoma – variant not associated with molecular identifiers, IDH2-mutated variant, HPV-related variant	‘Wastebasket’ entity grouping diverse tumors; includes variants with molecular identifiers (see also next row) Quick kinetic Knowledge, treatment, and prognosis have been changing over the last 3 decades Moderate-to-high risk of nodal metastasis^a^	Intermediate-to-high	nCT + (C)RT nCT + S + (C)RT
Sinonasal undifferentiated carcinoma – SMARCB1/INI1-deficient variant, SMARCA4-deficient variant	Very aggressive tumor Extremely quick kinetic High risk of nodal metastasis^a^ High risk of distant metastasis^a^	Very low	S + (C)RT nCT + (C)RT nCT + S + (C)RT
NUT carcinoma	Considered by some authors as the most dedifferentiated type of squamous cell carcinoma Very aggressive tumor Extremely quick kinetic High risk of nodal metastasis^a^ High risk of distant metastasis^a^	Very low	S + (C)RT nCT + (C)RT nCT + S + (C)RT
Sinonasal neuroendocrine carcinoma	Group of diverse carcinomas Might be found in combination with squamous cell carcinomatous or adenocarcinomatous tumor cells Generally, very aggressive tumor Quick kinetic High risk of nodal metastasis^a^ High risk of distant metastasis^a^	Low-to-very-low	S + (C)RT nCT + (C)RT nCT + S + (C)RT
Soft tissue sarcomas and borderline mesenchymal tumors – Rhabdomyosarcoma	Aggressive tumor Quick kinetic Subtype- and age-dependent behavior High risk of nodal metastasis^a^ High risk of distant metastasis^a^	Intermediate-to-low	S + (C)RT CRT + S nCT + S + (C)RT nCT + (C)RT + S
Soft tissue sarcomas and borderline mesenchymal tumors – Nonangiosarcoma, nonrhabdomyosarcoma	Group of diverse tumors Low risk of nodal metastasis^a^ Intermediate risk of distant metastasis^a^	Intermediate-to-high	S S + RT
Chondrosarcoma	Locally aggressive tumor Grade-dependent behavior Low risk of nodal metastasis^a^ Low risk of distant metastasis^a^	High-to-very high	S S + RT^c^ RT^c^
Osteosarcoma	Locally aggressive tumor Grade-dependent behavior Low risk of nodal metastasis^a^ Intermediate-to-high risk of distant metastasis^a^	Intermediate-to-high	S S + RT^c^ nCT + S + aCT nCT + S + RT nCT + S + RT + aCT RT^c^

a Refers to a rough estimate of the risk cumulating rate at presentation and recurrence (low: <10%; moderate: 10–20%; high: >20%).

b Refers to a rough estimate of the 5-year overall survival rate based on literature analysis and authors’ personal experience (very low: 0–20%; low: 20–40%; intermediate: 40–60%; high: 60–80%; very high: 80–100%).

c RT preferentially delivered with proton or carbon-ion radiotherapy. aCT, adjuvant chemotherapy; C, concomitant chemotherapy; HPV, human papillomavirus; nCT, neoadjuvant chemotherapy; OS, overall survival; RT, radiotherapy; S, surgery.

As aforementioned, the modern decision-making process to select the most appropriate surgery for a given sinonasal cancer starts by evaluating whether or not the lesion can be adequately resected through an endoscopic transnasal approach. Table 2 summarizes the most relevant topographical extensions that should be considered when selecting through which approach the tumor should be resected.

**Table 2:** Recommended surgery based on a topographical extension of the tumor

Local extension	Recommended surgical ablation	
Nasal septum, ethmoidal complex, anterior sphenoidal wall, sphenoidal floor, nasopharynx, medial maxillary wall, pterygopalatine fossa, infratemporal fossa (moderate invasion), upper parapharyngeal space, medial orbital bony wall, periorbit, extraconal fat (minimal invasion), medial wall of the lacrimal sac, nasolacrimal duct	Endoscopic resection	
Bony skull base (ethmoidal roof, cribriform plate, planum sphenoidale, tuberculum sellae, anteroinferior sellar wall, clivus), adjacent dura mater, falx cerebri (minimal macroscopic invasion), brain (minimal macroscopic invasion)	Endoscopic resection with transnasal craniectomy w/o subpial dissection	
Falx cerebri (nonminimal invasion), brain (nonminimal invasion), orbital roof, supraorbital dura	Cranioendoscopic resection	
Extraconal fat (nonminimal invasion), ocular muscles, eye, preseptal structures, orbital apex, lateral wall of the lacrimal sac	Orbital exenteration/clearance	
Nasal bones, frontal process of the maxillary bone, external nose	Partial or total rhinectomy	
Hard palate, inferior alveolar ridge	Open maxillectomy	Inferior maxillectomy
Maxillary sinus lumen (with no invasion of the orbital floor)		Subtotal maxillectomy
Orbital floor (even if with periosteum or extraconal fat minimal invasion)		Total maxillectomy w/o resection of the periorbit and inferior extraconal fat
Premaxillary periosteum, subcutaneous tissue, skin		Maxillectomy with resection of premaxillary soft tissues and/or rhinectomy
Buccal space, masticatory space		Maxillectomy extended to the infratemporal fossa
Frontal sinus lumen, anterior frontal plate, prefrontal soft tissues	Riedel's operation w/o resection of prefrontal soft tissues and/or rhinectomy	
Posterior frontal plate	Osteoplastic flap approach or Riedel's operation with posterior frontal craniectomy	

Postoperative RT has a well-established role in the treatment of most resectable craniofacial malignancies. So far, IMRT techniques represent the most frequently used RT approaches even if the literature on the efficacy of PT and CIRT is relentlessly growing, despite there is a paucity of particle therapy facilities worldwide. No guidelines are available to help clinicians in the choice between IMRT and particle therapy, particularly as regards PT. For nonradioresistant or relatively radioresistant tumors, such as SCC, SNUC, and neuroendocrine sinonasal carcinomas, for which the first goal is to reduce the risk of neurological radiation-induced adverse effects while achieving similar tumor control as compared to IMRT, a normal tissue complication probability (NTCP) model-based approach could be pursued [37]. This model-based selection is aimed to identify patients who are expected to benefit the most from PT. For radioresistant tumors, in particular in the case of gross residual disease after surgery, the use of PT and CIRT is strongly recommended in view of the capability of escalating the dose toward the target while minimizing radiation to neurological structures. This frequently applies to chordoma, chondrosarcoma, mucosal melanoma, and adenoid cystic carcinoma. All these considerations can be translated to the radical setting, when surgery is contraindicated.

## SYSTEMIC TREATMENTS

In light of the unsatisfactory results obtained in many locally advanced SNCs, resorting to systemic treatments is the logical step to improve prognosis in these diseases, thus, building on a multimodal approach typical of head and neck cancer management. However, data about the integration of systemic therapies in SNCs are scant, due to the rarity and heterogeneity of this disease and to the exclusion of this subsite in head and neck clinical trials assessing the role of chemotherapy, targeted agents, and immunotherapy. Even in recently published trials with immunotherapy in recurrent/metastatic setting and in ongoing trials where immune checkpoint inhibitors are administered concurrently and/or after radiation in locally advanced head and neck cancers, SNCs have not been included. Therefore, many of the assumptions about the role of systemic therapies derive from retrospective analysis or from transposing the ways of using systemic therapies in other subsites of the head and neck to SNCs.

With all these premises, one of the most promising uses of chemotherapy in SNCs is in an induction/neoadjuvant setting, with the aim of increasing locoregional control and reducing distant metastasis. Recently, Amit *et al.*[38^▪▪^] presented retrospective data of 95 patients with SNUC treated with induction chemotherapy followed by chemoradiation or surgery plus chemoradiation. They showed that response to induction chemotherapy determined the prognosis according to the following locoregional treatment received. In fact, in case of partial/complete response, patients who then received chemoradiation obtained more favorable 5-year disease-specific survival than those who received surgery followed by adjuvant (chemo)radiation (84% versus 51%); on the opposite, patients with stable disease or progression after induction chemotherapy had 5-year disease-specific survival of 0% with subsequent chemoradiation versus 39% with surgery and adjuvant (chemo)radiation.

At the moment, there are two ongoing trials exploring the activity and safety of induction chemotherapy in SNCs. The first one, led by ECOG-ACRIN, randomizes patients to neoadjuvant chemotherapy followed by surgery and postoperative RT versus surgery and postoperative RT for locally advanced nasal and paranasal sinus SCC (NCT03493425). The second one is a single-arm trial of induction therapy with docetaxel, cisplatin, and fluorouracil in locally advanced SCC and/or poorly differentiated carcinoma of the nasal cavity or paranasal sinuses (NCT00707473). Recently, two phase II trials of induction chemotherapy followed by surgery or (chemo)radiation with IMRT or heavy-ion radiation have closed their accrual (NCT02099175 and NCT02099188).

In this regard, the definition of predictors of response to induction chemotherapy is the logical step for a personalized approach. Recently, in a group of 13 patients with SNUC, 34 differentially expressed genes were identified as potentially distinguishing diseases achieving or not a response to induction chemotherapy [39]. The corresponding pathways involved the immune system, cell–extracellular matrix interaction, PI3K signaling, cell cycle, and apoptosis. In addition, the first data about radiomic in predicting the response of SNCs to induction chemotherapy are emerging. In a recent paper, Bologna *et al.*[40^▪^] showed how the apparent diffusion coefficient-based radiomic analysis could discriminate response to chemotherapy in a group of 50 patients with SNCs. Another field deserving to be studied is the need to add chemotherapy concurrently to radiation in high-risk SNCs. The ongoing GORTEC 2016-02 SANTAL trial (NCT02998385) is randomizing patients to receive or not cisplatin concurrent to radiation (IMRT or PT) for rare head and neck cancers, including sinonasal cancers.

## CONCLUSION

Treatment of SNCs has improved in the past years, thanks to the contribution of refined surgical indications and methods, better RT techniques, and better integration of systemic therapies. Similarly, diagnostic procedures give a better definition of the disease and support multidisciplinary group management.

As SNCs are rare and heterogeneous diseases, one should encourage small clinical trials with innovative statistical designs and with clear endpoints to be performed in multicenter settings. Every effort should be made to support multicenter and international cooperation, both to perform clinical trials and to reinforce translational research in this cancer type.

## Acknowledgements

*None.*

### Financial support and sponsorship

*AIRC Project No. 17422 – IG 2015 to P.B.*

### Conflicts of interest

*P.B. Advisory board or conference honoraria: Merck, Sanofi, Merck Sharp & Dohme, Sun Pharma, Angelini, Molteni, Bristol-Myers Squibb, Helsinn, GSK. M.F and E.O. do not have any conflicts of interest to declare.*

## REFERENCES AND RECOMMENDED READING

Papers of particular interest, published within the annual period of review, have been highlighted as:

▪ of special interest
▪▪ of outstanding interest
